# Age-related changes in visual encoding strategy preferences during a spatial memory task

**DOI:** 10.1007/s00426-021-01495-5

**Published:** 2021-03-23

**Authors:** Vladislava Segen, Marios N. Avraamides, Timothy J. Slattery, Jan M. Wiener

**Affiliations:** 1 Ageing and Dementia Research Centre, Bournemouth University, Bournemouth, UK; 2 Department of Psychology, Bournemouth University, Bournemouth, UK; 3 Department of Psychology, University of Cyprus, Nicosia, Cyprus; 4 CYENS Centre, Nicosia, Cyprus; 5 Aging and Cognition Research Group, German Center for Neurodegenerative Diseases (DZNE), Magdeburg, Germany

## Abstract

Ageing is associated with declines in spatial memory, however, the source of these deficits remains unclear. Here we used eye-tracking to investigate age-related differences in spatial encoding strategies and the cognitive processes underlying the age-related deficits in spatial memory tasks. To do so we asked young and older participants to encode the locations of objects in a virtual room shown as a picture on a computer screen. The availability and utility of room-based landmarks were manipulated by removing landmarks, presenting identical landmarks rendering them uninformative, or by presenting unique landmarks that could be used to encode object locations. In the test phase, participants viewed a second picture of the same room taken from the same (0°) or a different perspective (30°) and judged whether the objects occupied the same or different locations in the room. We found that the introduction of a perspective shift and swapping of objects between encoding and testing impaired performance in both age groups. Furthermore, our results revealed that although older adults performed the task as well as younger participants, they relied on different visual encoding strategies to solve the task. Specifically, gaze analysis revealed that older adults showed a greater preference towards a more categorical encoding strategy in which they formed relationships between objects and landmarks.

**Supplementary Information:**

The online version contains supplementary material available at 10.1007/s00426-021-01495-5.

## Introduction

Successful navigation and orientation depend on our ability to recognise familiar places across different perspectives (Waller & Nadel, 2013). In the lab, this ability is typically assessed with tasks in which participants first encode an array of objects or environmental features from one perspective and are then asked to indicate whether the array has changed when presented from a different perspective. Studies using such paradigms have reported age-related declines in performance (Hartley et al., 2007; Hilton et al., 2020; Montefinese et al., 2015; Muffato et al., 2019; Segen et al., 2020). Building on these studies, and to gain a more detailed understanding of the factors that contribute to the performance decline, we use eye-tracking to investigate potential age-related differences in visual encoding strategies. Specifically, we are interested in whether young and older adults rely on the same or different environmental cues during place recognition.

Recently, Muffato et al. (2019) and Hilton et al. (2020) investigated the effects of cognitive ageing on place recognition abilities using scenes defined by objects that were placed in an open field. After encoding a scene with four objects, participants were presented with another scene from a different perspective and had to decide whether or not it was identical to the one encoded. Results revealed the presence of object-location binding errors, particularly in older adults. That is, compared to younger participants, older adults found it harder to detect that two objects had swapped locations than when one of the objects was replaced with a new object.

In our previous work (Segen et al., 2020), we investigated age-related differences in the ability to recognise spatial configurations across different perspectives. The task required participants to encode the locations of an array of identical objects presented as an image on a computer screen. The objects were arranged in clusters of one, two and three objects, in a virtual room containing additional environmental cues such as windows and a door. Then, participants viewed a second image of the same room taken from the same (0°) or a different perspective (45° or 135°) and judged whether or not the objects were in the same locations. The positions of the objects were either changed by swapping two object clusters or by rotating one of the clusters. While with the former manipulation the task could be solved using a coarse categorical representation of the spatial relationships between object clusters (e.g. the cluster with two objects is to the left of the single object), the latter manipulation required a fine-grained spatial representation of the exact positions of the objects as the overall relationships between the clusters was maintained.

Consistent with previous research, we found that older adults had greater difficulty with the task than younger adults (Hartley et al., 2007; Hilton et al., 2020; Montefinese et al., 2015; Muffato et al., 2019). Diffusion modelling showed that older adults not only had greater difficulty in extracting useful information from the stimuli but that they also adopted a more conservative response strategy, i.e. they accumulated more information before reaching a decision.

Furthermore, the analysis of gaze data in Segen et al. (2020) revealed that older adults attended to a larger proportion of the scenes compared to younger adults. We proposed two potential explanations for this. First, differences in gaze behaviour may reflect differences in encoding strategies with older adults encoding object locations relative to the landmarks available in the room (windows, door, etc.), whilst young adults focus on the local arrangement of objects and on encoding the spatial relationships among them. The differences in encoding strategies may reflect a shift towards categorical spatial representations in older adults, driven by age-related hippocampal neurodegeneration (Antonova et al., 2009; Meulenbroek et al., 2004; Moffat et al., 2007).

Second, older adults may have difficulties in focusing on the task-relevant information as they become distracted by salient features within the environment. This is in line with the attention inhibition deficit in ageing reported in past studies (e.g., Hasher & Zacks, 1988). According to this account, older adults exhibit top-down control difficulties, with attention orienting being more affected by stimulus properties rather than the task at hand (Olk & Kingstone, 2015; West, 1996). Lastly, older adults may have difficulties in selecting the appropriate information required to solve the task. This is consistent with our findings that older adults have difficulties in extracting useful information from the stimuli (Segen et al., 2020).

In our earlier study (Segen et al., 2020), we could not distinguish between these explanations for several reasons. First, we did not systematically manipulate the availability of landmarks. Second, the landmarks used in the environment were all unique and informative and could, therefore, facilitate the encoding of the object locations, even if they distracted the older participants. Third, there was substantial overlap between the landmarks and the objects of the scene, which prevented a region of interest analysis. Finally, due to the large perspective shifts introduced in some trials (e.g. 135°), some landmarks were visible during encoding but not at test.

The current study was designed to disentangle the explanations for age-related differences in place recognition by examining gaze behaviour. To do so, we amended our original task (Segen et al., 2020) in a variety of ways to overcome the limitations of the earlier study. First, we reduced the size of the perspective shift between encoding and test which allowed us to present the same landmarks during learning and test, ensuring that participants could use the information they encoded during learning to solve the task at test. Decreasing the size of the perspective shift also made the task easier (Hegarty & Waller, 2004; Montofinese et al., 2015; Segen et al., 2020; Muffato et al., 2019; Hilton et al., 2020). Task difficulty was further reduced by including only the condition in which two object clusters were swapped with each other. Reducing task difficulty aimed at avoiding floor level performance in older adults, which would allow us to rule out that potential differences in gaze behaviour across groups are caused by participants’ inability to carry out the task.

Generally, we predict a decline in performance in older adults consistent with age-related place recognition deficits (Hartley et al., 2007). Responding after a perspective shift requires additional and demanding mental manipulations of the stored representations (e.g., mentally rotating the new or the stored representation to match the other, imagining moving around the array; Hegarty & Waller, 2004; Holmes et al., 2018; King et al., 2002). Therefore, we expect that the introduction of the perspective shift would impair performance in both groups. However, we predict a larger decrease in performance in older adults who seem to have difficulties with initiating those mental manipulations as reflected in past findings documenting larger impairments with the introduction rather than the increase of the perspective shift (Montofinesse et al., 2015; Muffato et al., 2019; Hilton et al., 2020; Segen et al., 2020).

To investigate the role of landmarks in encoding strategies and performance, we included trials in which landmarks (in the form of posters on the walls) were: (1) unique and could be used to encode object locations, (2) identical and thus uninformative or (3) absent from the scene. Varying the availability and utility of room-based landmarks allowed us to test whether age-related differences in gaze behaviour during spatial encoding were due to older adults encoding object positions by relating them to the landmarks or to older adults having difficulties in selecting and/or focusing on task-relevant information.

Since this part of the study is largely exploratory, we have formulated a series of predictions about results that we would expect to find depending on how older adults use additional landmarks during encoding of object locations. Given that the task can be solved either by focusing on the local arrangement of objects or by relating object positions to landmarks, we should not necessarily expect age-related differences in performance if older adults simply shift towards a particular encoding strategy depending on which information is available. However, if older adults select an encoding strategy that depends on the availability of landmarks as suggested by our previous research (Segen et al., 2020), we expect them to perform better when landmarks are informative than uninformative. Finally, if older adults have difficulties focusing on task-relevant information as a result of an attention inhibition deficit (Hasher & Zacks, 1988), and are therefore distracted by the presence of landmarks, we predict worse performance when landmarks are available (either informative or uninformative) than when they are not.

In terms of gaze behaviour, if older adults rely more on landmarks as part of their encoding strategy, compared to their younger counterparts, we expect them to spend more time gazing at informative landmarks than uninformative landmarks. If, however, older adults are distracted by the landmarks, we expected them to show similar gaze behaviour in conditions with informative and uninformative landmarks.

## Method

### Participants

Twenty-eight young (mean age = 21.00 years, SD = 2.27; age range = 18–27 years; 15 females and 13 males) and 32 older adults aged 60 years and over (mean age = 68.80, SD = 6.34, age range = 60–85; 17 females and 15 males) took part in this study. Participants were recruited either through the participant recruitment system of Bournemouth University or through opportunity sampling in the community. Older adults received monetary compensation for their time whilst younger participants received course credits. Participants were screened for mild cognitive impairment using the Montreal Cognitive Assessment (MoCA; Nasreddine et al., 2005). Based on a threshold score of 23, no participants were excluded (Luis et al., 2009; Waldron-Perrine & Axelrod, 2012). All participants gave their written informed consent in accordance with the Declaration of Helsinki (World Medical Association 2013).

### Virtual environment

**Fig. 1:**
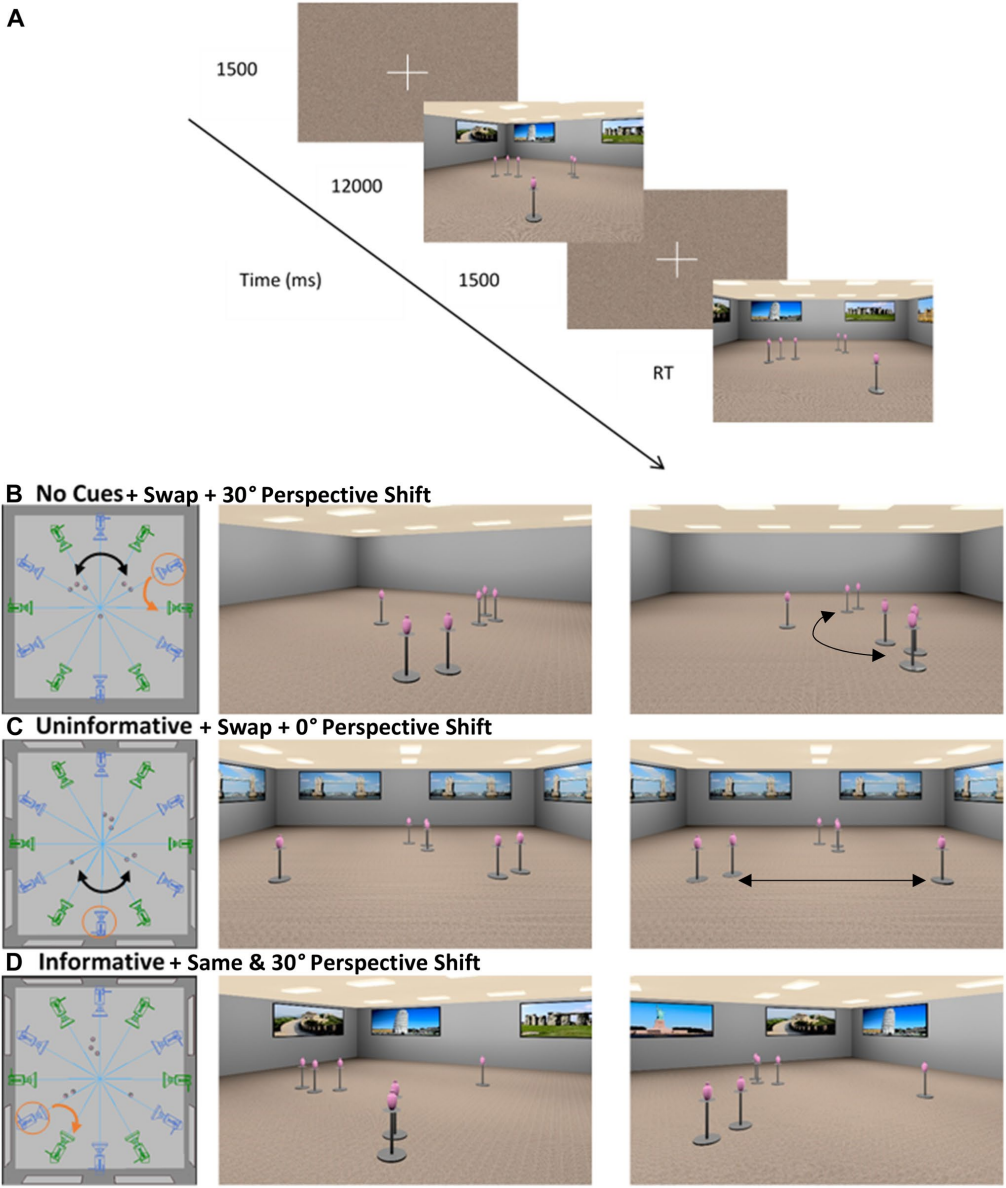
**a** Experimental protocol; **b**, **c** and **d** Virtual environment and stimuli for the experimental task, Blue and Green cameras represent the possible virtual cameras positions for the Learning and Test phase, respectively. Examples of possible object cluster layouts are shown in **b** (*No Landmarks*), **c** (*Uninformative Landmarks*) and **d** (*Informative Landmarks*). The *left panel* shows a survey perspective of the example trials, indicating the rotation of the camera (*Orange arrow*) and swapping of the two object clusters (*Black arrow*) in *Swap* trials (**b**, **c**). The *middle* and *right panels* show the two corresponding snapshots for the learning and test phases, respectively. In **b** and **d** there is a 30° perspective shift, to the left and right respectively. In **c** there is no perspective shift. The black arrows in the right panel (**b**, **c**) indicate which clusters were swapped on the test stimuli

The virtual environment was designed with Adobe 3DS Max 2018 and depicted a 13.5 m × 14.6 m rectangular room. The room contained 6 identical objects; pink vases on metal stands that were arranged in three clusters of 1, 2 and 3 objects in the centre of the room (see Fig. 1). In the *No Landmarks* condition, the walls contained no additional cues, in the *Uninformative Landmarks* condition eight identical posters of the Tower Bridge were presented, two on each wall. Finally, in the *Informative Landmarks* condition eight unique posters were presented, again two on each wall. These posters consisted of highly familiar and recognisable landmarks (Hamburger & Röser, 2014): the Leaning Tower of Pisa, Stonehenge, the Statue of Liberty, the Golden Gate Bridge, the Eiffel Tower, the White House, the Big Ben, and the Great Wall of China.

The experimental stimuli were renderings of the environment with a 70° horizontal field of view (FOV) with a 15% downward shift in the vertical FOV, yielding an asymmetric viewing frustum to simulate human vision. The virtual cameras from which the static images of the scenes were rendered were arranged on a circle (radius of 6.7 m) at 30° intervals, providing 12 possible camera positions and the object clusters were arranged in six unique layouts within the room (Fig. 1b-d). Six of those camera positions were used in the learning phase and in the *0°* perspective shift condition. The remaining 6 viewpoints were used in the test phase in the *30°* perspective shift condition. Stimuli were presented as static images on a desktop computer with OpenSesame 3.1.7 (Mathôt et al., 2012) and a standard computer keyboard was used to collect responses.

### Eye-tracking

Eye movements were recorded using an Eyelink II (SR Research) head-mounted eye tracker at a rate of 500 Hz. Calibrations were performed at least three times and drift correction was performed before each trial. The experiment was presented on a 102 cm screen (diagonal) with an aspect ratio of 16:9 and a resolution of 1920 × 1080 pixels. Participants were seated 100 cm from the monitor resulting in a physical horizontal FOV of 47.9° and 28° vertical FOV.

### Procedure

Each experimental trial started with a fixation cross and a scrambled stimuli mask presented for 1500 ms (Fig. 1a). In the learning phase, participants were presented with a rendering of one of the 6 unique configurations of the target objects from one of the six possible viewpoints for 12 s. After this learning phase, participants were again presented with a fixation cross and a scrambled stimuli mask for 1500 ms. Then, in the test phase they were presented with a rendering of the room either from the same viewpoint (50% of trials, Fig. 1c) or a different viewpoint that was offset by 30° from the study viewpoint (Fig. 1b, d). Participants were asked to respond by pressing the x or m keys on the keyboard as to whether the target objects were in the same locations as during the training phase or not. In 50% of the trials, the target objects remained in the same locations (*Same,* Fig. 1c) and in the other 50% of the trials, two of the three object clusters swapped locations (*Swap,* Fig. 1b, c). As a result, chance level performance for this task was 50%.

The experiment consisted of 144 experimental trials that were preceded by 6 practice trials. The entire study took around 90 min to complete and participants were allowed to take breaks when they wished.

### Design

The experiment followed a mixed 2 (Age Group: *young vs. older adults*) × 2 (Manipulation: *Same, Swap,* Fig. 1b,c and d) × 2 (Perspective Shift: *0°, 30°*) × 3 (Landmark Type: *No Landmarks, Uninformative, Informative*) design with Manipulation, Perspective Shift and Landmark Type manipulated within participants and Age Group between.

### Data Analysis

Data from one older participant were excluded from all analyses due to chance level performance in the 0° Perspective Shift condition. The remaining data were analysed with linear mixed-effects models (LME) using LME4 (Bates et al., 2018) in R (R Core Team, 2013). Specifically, accuracy was analysed using generalized linear mixed-effects (GLME) models with the *glmer* function from LME4 package. The following contrasts were used in all (G)LMEs conducted: Age Group (*Younger adults/Older adults*), Perspective shift (0°/30°) and Manipulation (*No Change/Swap*) were coded using effect coding. This coding scheme compares the effect of a variable (i.e. Age Group) on performance averaged across all levels of other variables (i.e. Perspective Shift and Manipulation). Landmark Type was coded using treatment coding. Since we were interested in examining the difference between *Informative* and *Uninformative Landmarks* and the difference between *No Landmarks* and *Uninformative Landmarks,* we used the *Uninfomative Landmark* as the baseline. As a result, all of the effects for other factors are calculated with reference to the performance in the *Uninformative Landmark,* rather than the average of performance for all levels of Landmark Type. For the response time analysis, we included only the correct trials and we log-transformed response times following the recommendations of Baayen et al. (2008) for dealing with the skewness of the response time distribution. Prior to transforming, response times below 200 ms and over 20,000 ms were removed.

## Results

### Accuracy

**Fig. 2:**
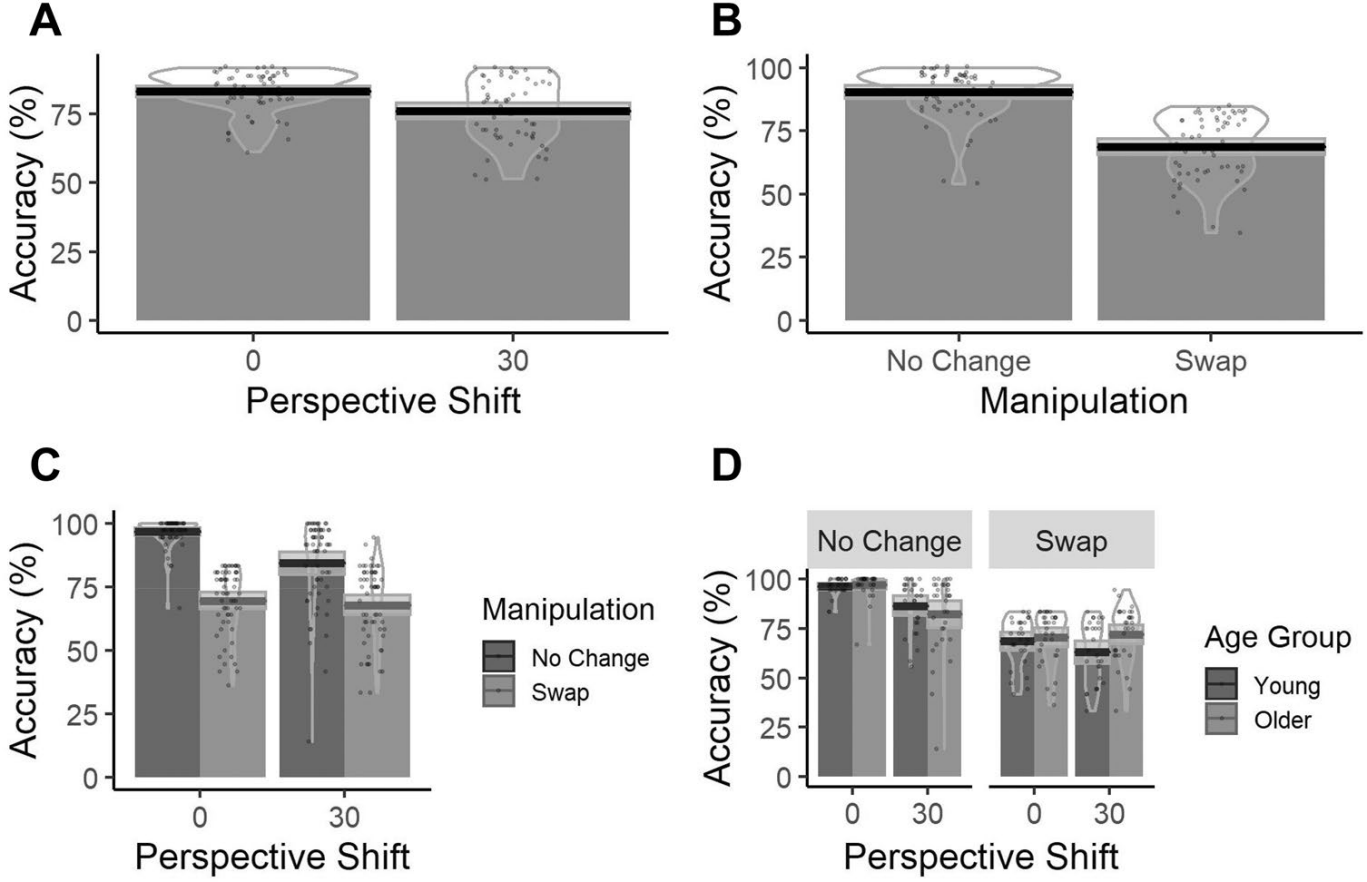
Bar plots of accuracy values for a significant main effect of **a** Perspective Shift, **b** Manipulation, and significant interactions **c** between Manipulation and Perspective Shift and **d** Interaction between Age Group, Manipulation and Perspective Shift with a mean (solid line) and 95% CIs (grey shaded area) with individual data points and violin plots behind

Accuracy estimates were obtained for each participant with Age Group, Perspective Shift, Landmark Type and Manipulation as fixed factors and a random by-subject and by-item intercept. Coefficients, standard errors and *z*-values (Table 1) indicate that Perspective Shift and Manipulation affected performance. Specifically, accuracy decreased with the introduction of a 30° Perspective Shift (Fig. 2a) and in the *Swap* condition (Fig. 2b). In addition, there was an interaction between Perspective Shift and Manipulation with a greater decline in performance in the *No Change* condition compared to the *Swap* condition following a 30° Perspective Shift (Fig. 2c). Finally, we found a three-way interaction between Perspective Shift, Manipulation and Age Group with older adults showing a larger decline in performance than younger adults in the *No Change* condition when a 30° Perspective Shift was introduced, whilst displaying an increase in performance in the Swap condition when a 30° Perspective Shift was introduced (Fig. 2d). Effect plots for significant main effects and interactions are reported in the Supplementary Materials.

**Table 1:** Coefficients from Accuracy GLME analysis

Predictors	Accuracy		
	Coefficients	Std. Error	*z*-value
(Intercept)	2.023	0.262	**7.724**
Age Group (*Old*)	0.145	0.112	1.293
Perspective Shift (*30°*)	− 0.635	0.079	**− 8.049**
Landmark Type (*Informative*)	− 0.122	0.347	− 0.350
Landmark Type (*No Landmarks*)	0.066	0.350	0.189
Manipulation (*Swap*)	− 1.316	0.086	**− 15.216**
Age Group (*Old*): Perspective Shift (*30°*)	− 0.104	0.071	− 1.468
Age Group (*Old*): Landmark Type (*Informative*)	− 0.063	0.095	− 0.659
Age Group (*Old*): Landmark Type (*No Landmarks*)	− 0.138	0.105	− 1.314
Perspective Shift (*30°*): Landmark Type (*Informative*)	0.176	0.106	1.659
Perspective Shift (*30°*): Landmark Type (*No Landmarks*)	− 0.037	0.116	− 0.319
Age Group (*Old*): Manipulation (*Swap*)	0.063	0.071	0.887
Perspective Shift (*30°*): Manipulation (*Swap*)	0.414	0.077	**5.387**
Landmark Type (*Informative*): Manipulation (*Swap*)	0.212	0.115	1.846
Landmark Type (*No Landmarks*): Manipulation (*Swap*)	− 0.082	0.125	− 0.656
Age Group (*Old*): Perspective Shift (*30°*): Landmark Type (*Informative*)	0.097	0.095	1.020
Age Group (*Old*): Perspective Shift (*30°*): Landmark Type (*No Landmarks*)	0.137	0.105	1.303
Age Group (*Old*): Perspective Shift (*30°*): Manipulation (*Swap*)	0.240	0.071	**3.399**
Age Group (*Old*): Landmark Type (*Informative*): Manipulation (*Swap*)	0.049	0.095	0.514
Age Group (*Old*): Landmark Type (*No Landmarks*): Manipulation (*Swap*)	0.054	0.105	0.512
Perspective Shift (*30°*): Landmark Type (*Informative*): Manipulation (*Swap*)	0.060	0.103	0.584
Perspective Shift (*30°*): Landmark Type (*No Landmarks*): Manipulation (*Swap*)	0.155	0.114	1.364
Age Group (*Old*): Perspective Shift (*30°*): Landmark Type (*Informative*): Manipulation (*Swap*)	− 0.122	0.095	− 1.277
Age Group (*Old*): Perspective Shift (*30°*): Landmark Type (*No Landmarks*): Manipulation (*Swap*)	− 0.201	0.105	− 1.916

Significant *z* values (|*z*|≥ 1.96) in bold

### Response Time

**Fig. 3:**
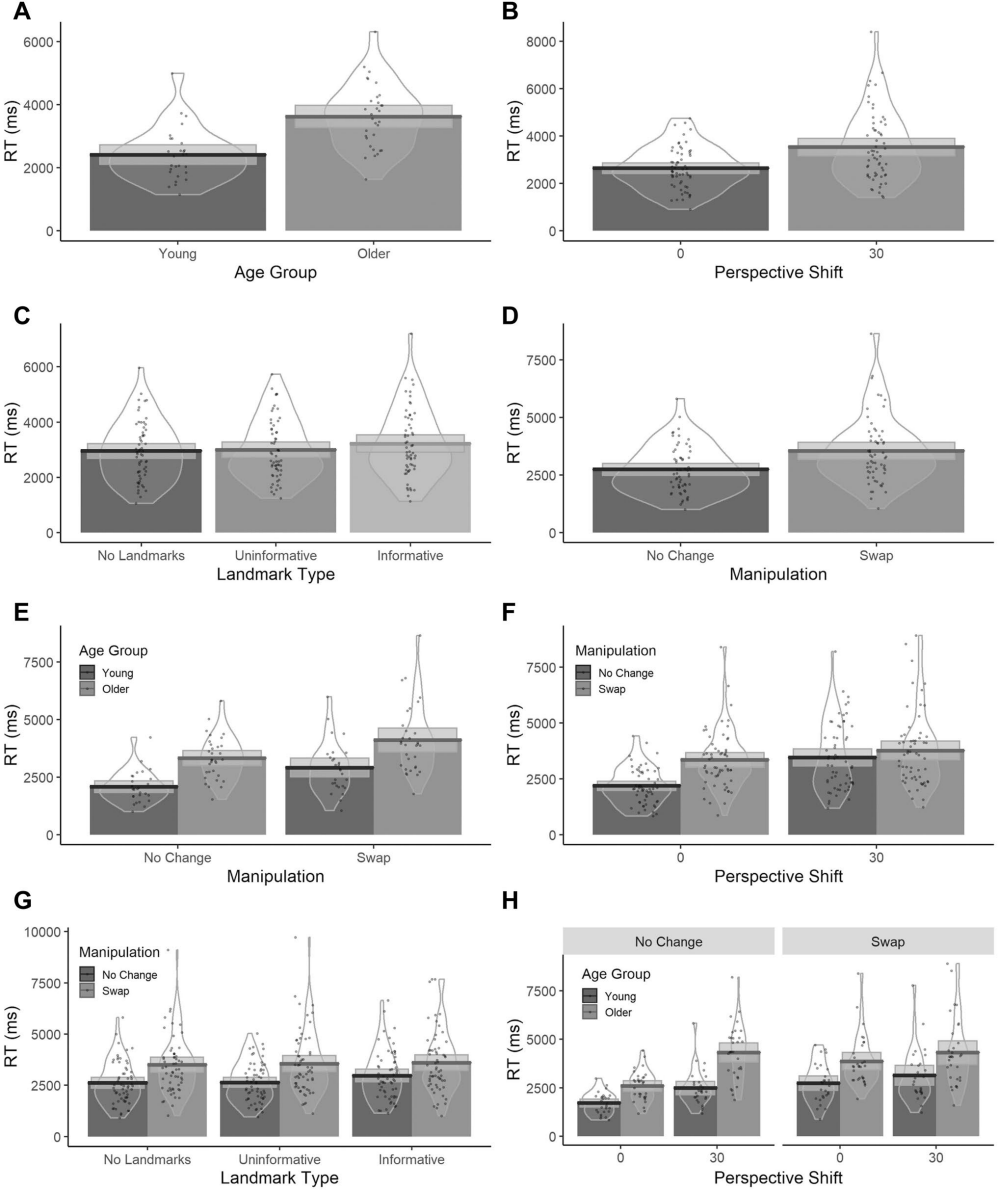
Bar plots of Response Times values for significant main effects of A: Age Group B: Perspective Shift C: Landmark Type (significant only for the *Informative* Landmark Type) D: Manipulation and interactions between E: Age Group and Manipulation F: Perspective Shift and Manipulation G: Landmark Type and Condition (significant only for Landmark Type (*Informative*): Manipulation (*Swap*)) H: Age Group, Manipulation and Perspective Shift with mean (solid line) and 95% CIs (grey shaded area) with individual data points and violin plots behind

As with accuracy, response time estimates were obtained for each participant with Age Group, Perspective Shift, Landmark Type and Manipulation as fixed factors and a random by-subject and by-item intercept with a random slope for Manipulation across participants. Coefficients, standard errors and *t*-values (Table 2) show that Age Group, Perspective Shift, Landmark Type and Manipulation were all reliable predictors of response time. Specifically, we found that older adults were slower to respond compared to younger adults (Fig. 3a), and that response times increased with the introduction of a Perspective Shift (Fig. 3b). In addition, response times were longer with *Informative* than *Uninformative* Landmark Type (Fig. 3c) and in the *Swap* condition compared to the *No Change* condition (Fig. 3d). We also found a significant interaction between Age Group and Manipulation with a smaller increase in response times in the *Swap* condition in older than younger adults (Fig. 3e). There was also a Perspective Shift and Manipulation interaction with a smaller increase in response times in the *Swap* condition than the *No Change* condition with the introduction of the Perspective Shift (Fig. 3f). We also found an interaction between Landmark Type and Manipulation with a smaller increase in response times between the *No Change* and the Swap condition in the *Informative* Landmark Type (Fig. 3g) compared to *Uninformative* Landmark Type condition. Finally, we found a three-way interaction between Age Group, Perspective Shift and Manipulation, with the Age Group and Perspective Shift interactions showing a different trend across *No Change* and *Swap* Manipulation. Specifically, there was a larger increase in response times in older adults than young adults, in the *No Change* condition with the introduction of the Perspective Shift (Fig. 3)*.* Whilst in the *Swap* condition, the increase in response times in older adults was smaller when a Perspective Shift was introduced compared to young adults. Effect plots for significant main effects and interactions are reported in the Supplementary Materials.

**Table 2:** Coefficients from response time LME analysis

Predictors	Log transformed response time		
	Estimates	Std. Error	*t*-value
(Intercept)	7.834	0.041	**190.067**
Age Group	0.209	0.040	**5.248**
Perspective Shift (*30°*)	0.130	0.015	**8.459**
Landmark Type (*Informative*)	0.058	0.020	**2.942**
Landmark Type (*No Landmarks*)	− 0.013	0.020	− 0.640
Manipulation (*Swap*)	0.133	0.011	**12.386**
Age Group: Perspective Shift (*30°*)	0.006	0.014	0.451
Age Group: Landmark Type (*Informative*)	0.019	0.013	1.470
Age Group: Landmark Type (*No Landmarks*)	− 0.019	0.013	− 1.443
Perspective Shift (*30°*): Landmark Type (*Informative*)	0.012	0.015	0.813
Perspective Shift (*30°*): Landmark Type (*No Landmarks*)	− 0.000	0.015	− 0.007
Age Group: Manipulation (*Swap*)	− 0.032	0.009	**− 3.474**
Perspective Shift (*30°*): Manipulation (*Swap*)	− 0.077	0.010	**− 7.542**
Landmark Type (*Informative*): Manipulation (*Swap*)	− 0.034	0.015	**− 2.259**
Landmark Type (*No Landmarks*): Manipulation (*Swap*)	0.010	0.015	0.654
Age Group: Perspective Shift (*30):* Landmark Type (*Informative*)	− 0.003	0.013	− 0.239
Age Group: Perspective Shift (*30°*): Landmark Type (*No Landmarks*)	− 0.013	0.013	− 1.012
Age Group: Perspective Shift (*30°*): Manipulation (*Swap*)	− 0.018	0.009	**− 1.960**
Age Group: Landmark Type (*Informative*): Manipulation (*Swap*)	− 0.008	0.013	− 0.596
Age Age Group: Landmark Type (*No Landmarks*): Manipulation (*Swap*)	− 0.024	0.013	− 1.847
Perspective Shift (*30°*): Landmark Type (*Informative*): Manipulation (*Swap*)	0.019	0.014	1.312
Perspective Shift (*30°*): Landmark Type (*No Landmarks*): Manipulation (*Swap*)	0.002	0.014	0.162
Age Group: Perspective Shift (*30°*): Landmark Type (*Informative*): Manipulation (*Swap*)	0.005	0.013	0.406
Age Group: Perspective Shift (*30°*): Landmark Type (*No Landmarks*): Manipulation (*Swap*)	− 0.004	0.013	− 0.289

Significant t values (|t|≥ 1.96) in bold

## Response Bias

To examine if participants displayed a response bias, we carried out an analysis based on Signal Detection Theory (Harvey, 1992; Macmillan & Creelman, 1991) using the sdt.rmcs (Todorova, 2017) package in R. Signal Detection Theory evaluates sensitivity and response bias in situations that require decision making under uncertainty. It is applied when a binary decision about the presence or absence of a signal is made, comparing the response with the actual presence/absence of the signal. With Signal Detection Theory, the formula c = *-0.5*[z(hit rate) + *z*(false alarm rate) is used to compute response bias, where hit rate and false alarm rates refer to trials in which the signal was correctly or incorrectly, respectively, reported as present.

**Fig. 4:**
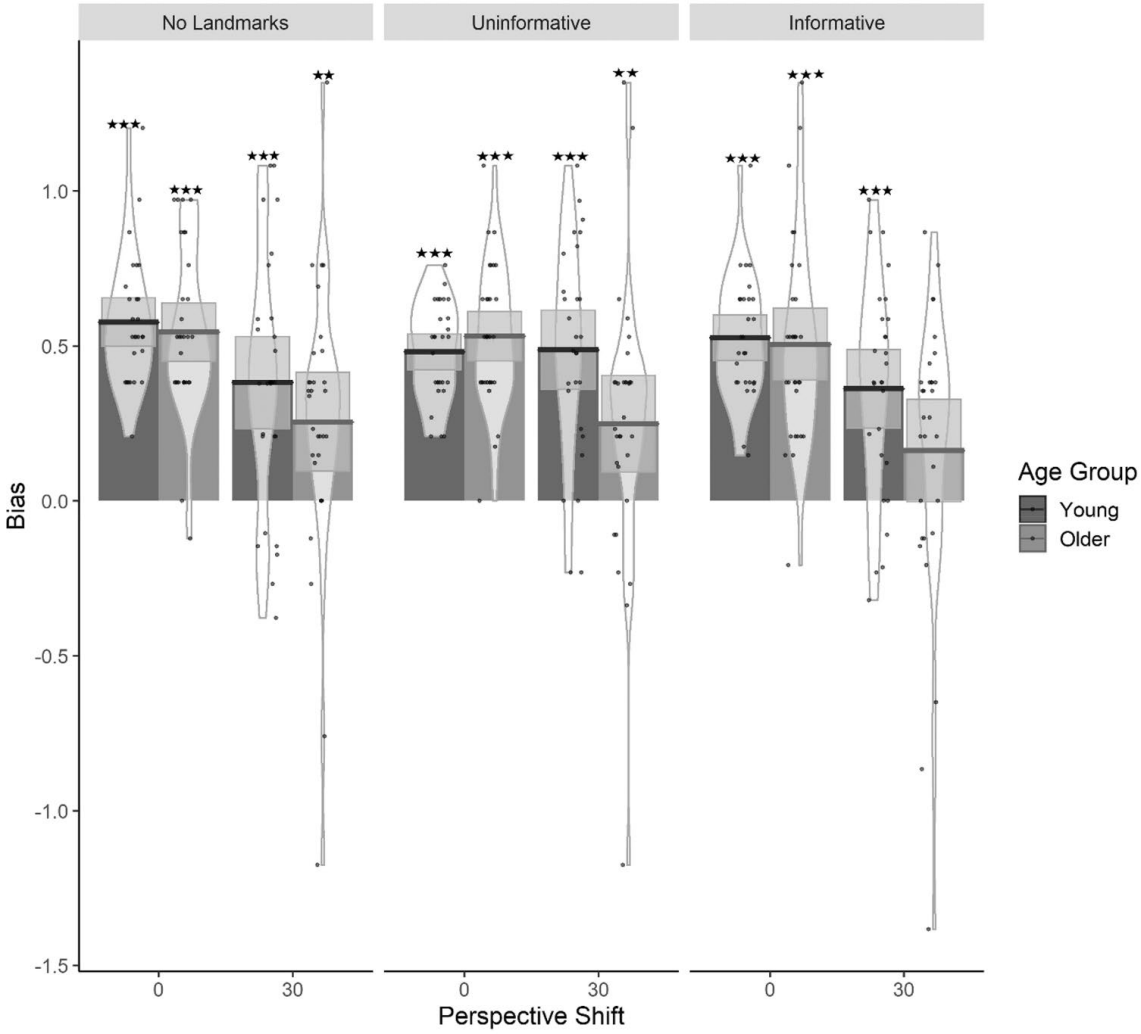
Bar plots for Response Bias as a function of Age Group, Landmark Type and Perspective Shift with mean (solid line) and 95% CIs (grey shaded area) with individual data points and violin plots behind. Stars indicate response bias significantly different from 0 (1 star [*p* < 0.05], 2 stars [*p* < 0.01] and 3 stars [*p* < 0.001])

Overall, there was a positive response bias showing that participants were more likely to respond that nothing has changed than to respond that something had changed (Fig. 4). LMM analysis (Table 3) with Age Group, Perspective Shift and Landmark Type as fixed factors and by-subject intercept with a random slope for Perspective Shift, indicated that the introduction of a Perspective Shift led to a decrease in response bias, which was larger in older adults than in younger adults. Furthermore, when a Perspective Shift was introduced, the response bias decreased more in the *No Landmarks* and the *Informative Landmarks* conditions compared to the *Uninformative Landmarks* condition (Table 4).

**Table 3:** Coefficients from Response Bias LME analysis

*Predictors*	Response bias (c)		
	Estimates	Std. Error	*t*-value
(Intercept)	0.437	0.033	**13.043**
Age Group (*Older Adults*)	− 0.047	0.033	− 1.403
Perspective Shift (*30°*)	− 0.069	0.029	**− 2.384**
Landmark Type (*Informative*)	0.003	0.026	0.097
Landmark Type (*No Landmarks*)	− 0.048	0.026	− 1.826
Age Group: Perspective Shift (*30°*)	− 0.072	0.029	**− 2.495**
Age Group: Landmark Type (*Informative*)	0.007	0.026	0.264
Age Group: Landmark Type (*No Landmarks*)	− 0.008	0.026	− 0.306
Perspective Shift (*30°*): Landmark Type (*Informative*)	− 0.052	0.026	**− 1.978**
Perspective Shift (*30°*): Landmark Type (*No Landmarks*)	− 0.058	0.026	**− 2.201**
Age Group: Perspective Shift (*30°*): Landmark Type (*Informative*)	0.049	0.026	1.845
Age Group: Perspective Shift (*30°*): Landmark Type (*No Landmarks*)	0.028	0.026	1.043

Significant *t* values (|*t*|≥ 1.96) in bold

**Table 4:** Coefficients from Dwell Time on the top IA LME analysis

Predictors	Dwell Time on Landmarks		
	Estimates	Std. Error	*t*-value
(Intercept)	13.054	1.503	8.684
Age Group (*Older Adults*)	2.99/	1.457	**2.058**
Landmark Type (*No Landmarks*)	− 8.108	0.644	**− 12.600**
Landmark Type (*Informative*)	9.540	0.644	**14.826**
Age Group (*Older Adults*): Landmark Type (*No Landmarks*)	− 1.804	0.375	**− 4.812**
Age Group (*Older Adults*): Landmark Type (*Informative*)	1.171	0.375	**3.124**

Significant *t* values (|*t*|≥ 1.96) in bold

### Gaze analysis

Fixations and saccades were identified using the SR Research algorithms and were pre-processed as follows: First, we removed fixations that contained a blink, fell outside of the screen boundaries or were shorter than 80 ms or longer than 1000 ms (Inhoff & Radach, 1998; Nuthmann, 2017). Finally, we removed saccades with maximum amplitudes (41.35°va) or velocities (1500°/s) larger than it should be possible based on the distance of the participant from the screen and the screen size.

The primary aim of the gaze analysis was to investigate age differences in encoding strategies and was therefore mainly focused on the analysis of gaze during the encoding phase. Analysis of differences in basic saccade and fixation parameters between young and older adults showed that during the 12 s encoding period, older adults made shorter and more frequent fixations as well as more frequent saccades. The results are reported in detail in the supplementary materials.

### Gaze on landmarks

**Fig. 5:**
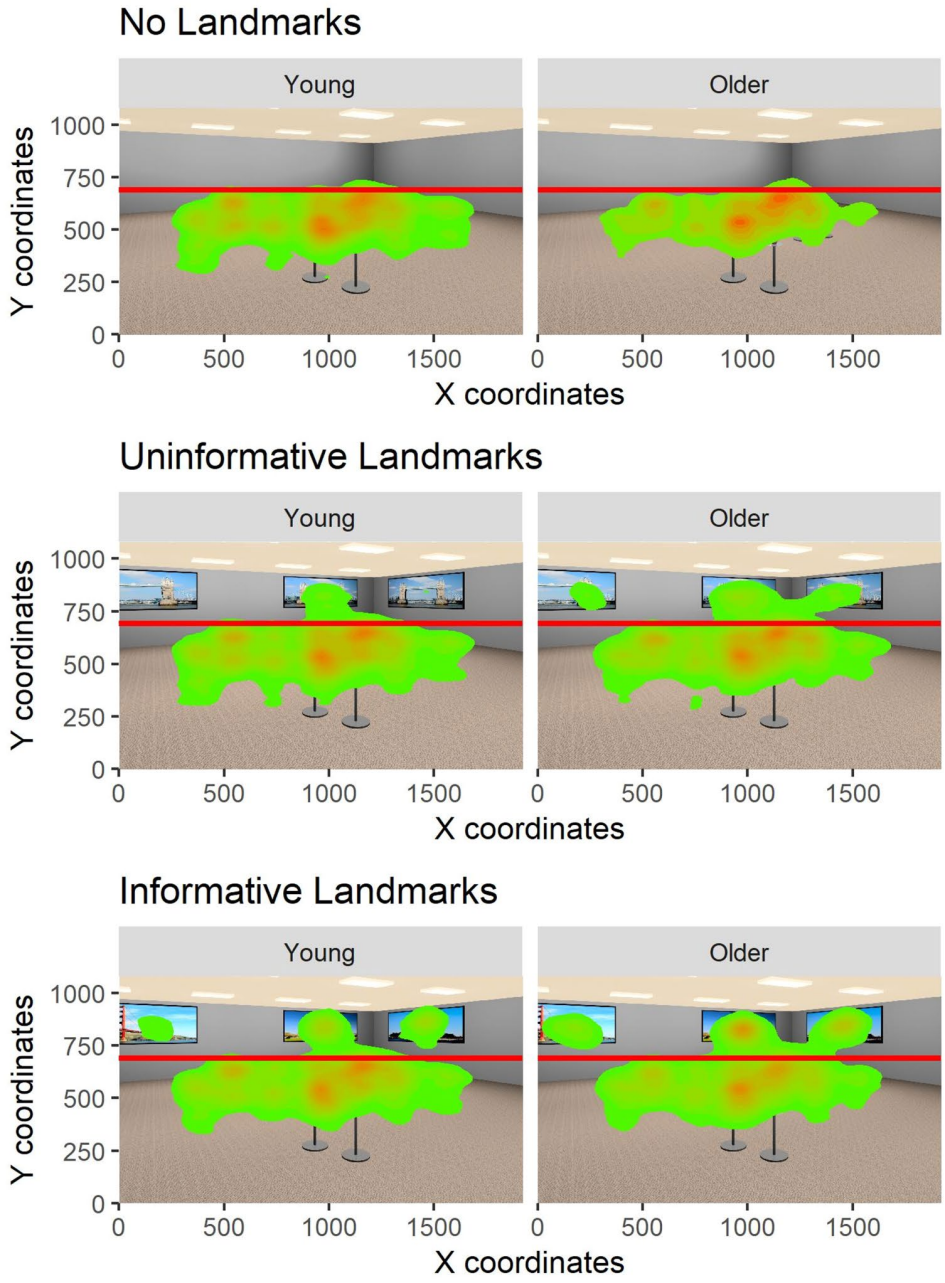
Heatmaps representing number of fixations as a function of age group and landmark type

As we were primarily interested in age-related differences in gaze as a function of Landmark Type, we split stimuli into two interest areas (See Fig. 5) and compared the percentage of Dwell Time on the top interest area (IA) where Landmarks were located when available vs. the bottom area where the objects were located. To do so, we computed the total dwell time for each trial by adding up the duration of all fixations in the trial. Next, we calculated the proportion of dwell time that was spent fixating in the top IA. This approach allowed us to specifically focus on age-related differences in the use of room-based Landmarks during encoding with the increased Dwell Time on the upper part of the stimuli largely reflecting gaze on Landmarks (when available).

LME analysis with Age Group and Landmark Type as fixed factors and a by-subject and by-item random intercept showed that Landmark Type and Age Group were reliable predictors of Dwell Time on the top IA. Specifically, we found that compared to the *Uninformative Landmarks* condition that was used as a baseline, there was a reduction in Dwell Time on the top IA in the *No Landmarks* and an increase in Dwell Time in the *Informative Landmarks* condition. We also found that older adults spent more time looking at the top IA compared to younger adults. In addition, there was a Landmark Type and Age Group interaction whereby older adults’ Dwell Time on Landmarks decreased more than that of younger adults’ in the *No Landmarks* condition compared to *Uninformative Landmarks* condition and showed a larger increase in the *Informative Landmarks* condition compared to the *Uninformative Landmarks* condition. A Dwell Time analysis on the top IA at test produced similar results to those of the learning phase, with the exception that the increase in Dwell Time in older adults and the Age Group by Landmark Type (No Landmarks) interaction were not significant. Results from this analysis are presented in the Supplementary Materials.

### Relationship between Gaze and Performance

**Fig. 6:**
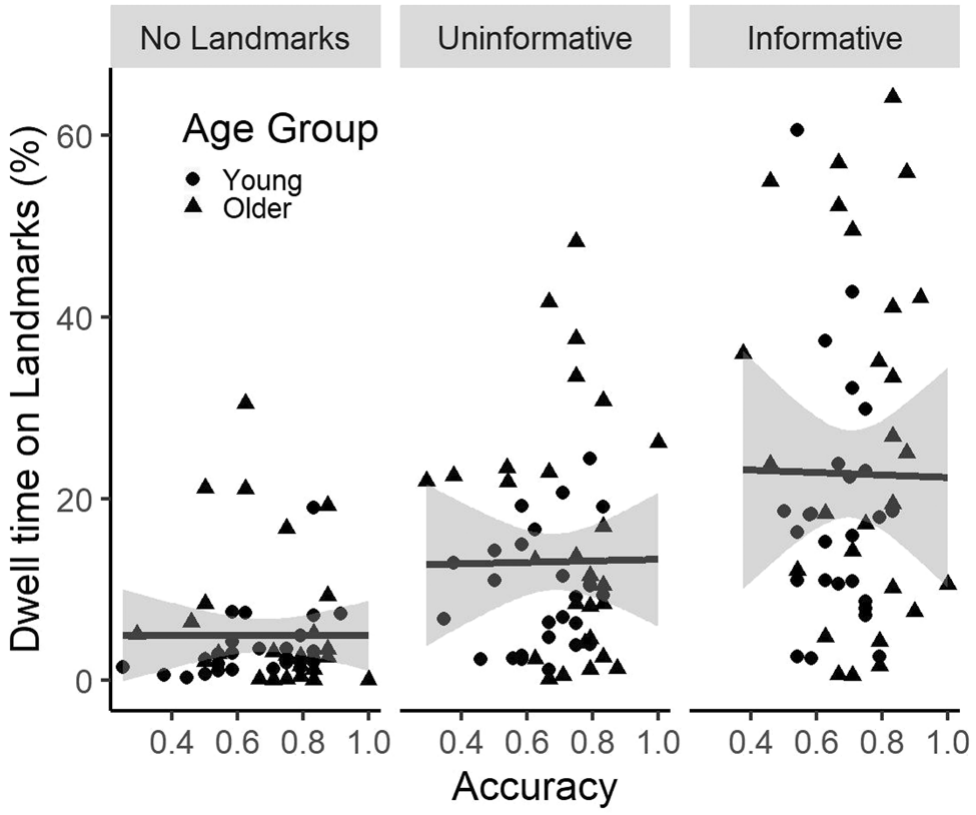
Scatter Plot between Dwell Time on the top IA and Accuracy as a function of Landmark Type with regression line and CI (shaded area)

Dwell time on the top IA was not related to performance across any of the three Landmark Type conditions (Fig. 6), suggesting that the task could be solved either by using Landmarks (when they are available) or by focusing primarily on the objects. Thus, the differences in gaze behaviour reported here are likely to represent differences in encoding strategy preferences that change with age.

### Gaze behaviour across trials

We also investigated if gaze behaviour changes across time by correlating Dwell Time on landmarks with trial older for younger and older participants in the *No* *Landmark*, *Uninformative* and *Informative* *Landmark* conditions. We found that across both younger and older adults, Dwell Time remained consistent in the *No Landmark* condition throughout the experiment (Young: *r* = 0.011, *p* = 0.895, Older: *r* = − 0.09, *p* = 0.279). In the *Uninformative Landmark* condition, older adults spent less time fixating on landmarks over the course of the experiment (*r* = − 0.18, *p* = 0.032), whilst younger adults' gaze (*r* = − 0.05, *p* = 0.543) remained unchanged. In the *Informative Landmark* condition, an opposite pattern of results was found with younger adults spending less time fixating on landmarks (*r* = − 0.20, *p* = 0.018) with older adults' gaze remaining unchanged (*r* = − 0.09, *p* = 0.266).

### Consistency in gaze between learning and test

Finally, we examined if participants showed similar gaze behaviour during learning and test. To do so, we correlated the Dwell Time on the top IA across different Landmark Types at learning and test. We found strong positive correlations across all Landmark Types *(No Landmarks*: R2 = 0.67, *p* < 0.001; *Uninformative*: R2 = 0.88, *p* < 0.001; *Informative*: R2 = 0.94, *p* < 0.001). Those correlations suggest that participants are highly consistent in which stimulus features they gaze at during encoding and test.

## Discussion

In the present study, we used eye-tracking to investigate age-related differences in visual encoding strategies employed for memorizing the locations of objects in a room. To do so, we explored if participants were able to identify whether a spatial scene has changed following a perspective shift between encoding and test. The 30*°* perspective shift was introduced to ensure that participants relied on spatial representations instead of solving the task by matching the visual image with a stored visual snapshot from encoding (Nardini et al., 2009). To investigate the effect of landmarks on encoding strategies, we also manipulated the availability and informative value of landmarks within the environment.

We found that overall, older adults took longer to respond. This increase in response times is consistent with findings that are widely reported in the cognitive ageing literature (Choice reaction time task: Woods et al., 2015; Memory: Hertzog et al., 2003; Language: Ratcliff et al., 2004a, b), and istypically attributed to decrements in speed of processing (Salthous, 1996; Salthouse & Ferrer-Caja, 2003). We also found that the introduction of the perspective shift and the manipulation of object positions led to performance decrements in both age-groups. The availability and informativeness of the room-based landmarks did not affect task accuracy. Importantly, we found that when landmarks were presented, older participants spent more time than younger participants looking at the upper part of the display that contained the landmarks. This was particularly the case when the landmarks were informative.

Contrary to our expectations and previous place recognition research (Muffato et al., 2019; Hilton et al., 2020; Segen et al., 2020; Harley et al., 2007), there were no age-related differences in accuracy. However, it should be noted that we used an easier task than those used in previous studies, which could yield fewer problems for older adults. For example, the perspective shift we introduced was smaller than that of previous studies (Muffato et al., 2019; Montofinese et al., 2015; Segen et al., 2020). In addition, the scene at test could differ from the encoded only in terms of a change in the categorical relationship between objects. That is, in contrast to Segen et al. (2020), in the current study no changes in fine-grained spatial relationships between objects occurred. That the easier task may be responsible for the lack of age-related deficits in task accuracy is in line with cognitive ageing research reporting greater age-related differences in performance with increasing task difficulty (Angel et al., 2016; Earles et al., 2004; Verhaeghen et al., 2006).

The lack of age-related performance accuracy differences in less demanding tasks can be explained by the compensation-related utilization of neural circuits hypothesis (Reuter-Lorenz & Cappell, 2008). This hypothesis posits that under low task demands older adults can perform the tasks as well as young adults, supported by increased neural activations. However, when task demands increase, older adults’ cognitive limits are reached resulting in performance declines that are typically accompanied by a reduction in activation in the relevant neural networks (Morcom & Rugg, 2007; Angel et al., 2016). Thus, it is plausible that due to the relatively low task-demands in the current study, which are reflected in high performance across both age groups, older adults were able to carry out the task just as accurately as younger participants.

Consistent with our predictions, we found declines in accuracy in both age groups that were accompanied by an increase in response times when a perspective shift was introduced. This reduction in performance may have been driven by qualitative differences between trials that involved a perspective shift and those that did not. Specifically, without a perspective shift participant can refer to the representation of the learned scene from memory and use image matching to detect changes (Nardini et al., 2009). However, the introduction of the perspective shift required participants to engage in additional cognitive processing related to mental transformation in order to match the perspectives of the stored spatial configuration with the one presented at test (Hegarty & Waller, 2004). However, it should be noted that the effect of the perspective shift was small, which is likely due to the relatively small perspective shift that we introduced.

Interestingly, there was a much more nuanced (if any) decline in accuracy or increase in response time in the *Swap* compared to the *No Change* condition when a perspective shift was introduced. To explain such findings, we turn to the response bias analysis which suggested that the introduction of the perspective shift increased the likelihood of participants responding that the object positions were “different”. Thus, when a perspective shift was introduced in the *Swap* condition, this led to an increase in the number of correct responses albeit for the wrong reason. We believe that the increase of “different” responses after a perspective shift arises from the salient change in the visual input indicating that “something is different”. However, if participants were solely responding to any change in the visual information between encoding and test, we expected them to perform below chance level in the *No Change* condition when a perspective shift was present. Yet, our participants were still able to perform well in this condition and their performance in the *Swap* condition with perspective shifts was not at the ceiling. This pattern of results demonstrates that participants were not solely relying on basic visual change detection but were instead using a spatial strategy to perform the task. Yet, they might have found it hard to inhibit the immediate response that the image is “not the same” when the perspective shift was introduced. The increase in performance in older adults with the introduction of the perspective shift in the *Swap* condition may thus be due to older adults experiencing even greater difficulty in inhibiting the response that the image is “not the same” when a perspective shift was present. Such difficulties are in line with age-related decline in executive functioning, in particular executive control (Braver & West, 2008; Schretlen et al., 2000; Treitz et al., 2007).

Overall participants were more likely to make errors in the *Swap* condition than the *No Change* condition. To perform the task accurately participants in either condition had to bind an object's identity to its location (Postma et al., 2004; Waller, 2006). Previous research has shown that this is a cognitively demanding and error-prone process. For example, in place recognition studies participants were shown to be less accurate in detecting that a change has occurred when two objects swapped places compared to when a previously shown object was replaced by a new one (Hilton et al., 2020; Muffato et al., 2019). Similar results are reported in visuospatial working memory studies in which participants were asked to encode positions of abstract objects on a blank display. Participants were more likely to make swap errors, that is to place objects in the positions that were previously occupied by a different object (Pertzov et al., 2012, 2015).

Thus, the lower performance in the *Swap* condition can be explained by difficulties with binding objects to their locations, which prevents participants from accumulating information signalling that a change has occurred (Hilton et al., 2020; Muffato et al., 2019). Specifically, in the current task, the objects within the scene and their general configuration remained the same between learning and test. The only change introduced in the *Swap* condition is the position that each cluster occupied within that general configuration. Therefore, participants needed to remember the specific locations of each object cluster within that configuration to detect that a change has occurred.

In addition to comparing the behavioural performance of older and younger adults, another aim of this study was to use eye-tracking to investigate age-related differences in spatial encoding strategies and to study if such differences are driven by the information available within the environment. Firstly, we focused on general gaze parameters and found that older adults made more fixations that were shorter in duration as well as shorter saccades than young adults. While these results are consistent with those from a recent study using a similar place recognition task (Hilton et al., 2020), relating these general gaze measures to encoding strategies is difficult. We thus performed IA analysis which showed that gaze behaviour differed as a function of room type. As expected, we found that both age groups spent the lowest amount of time looking at the upper part of the stimuli in the *No Landmarks* condition in which there were no images on the walls of the room, followed by the *Uninformative Landmarks* condition, in which the images on the walls were all identical, and the *Informative Landmarks* condition in which each image was unique. These findings are consistent with results reported by Livingstone-Lee et al. (2011) who showed that participants quickly learned to adapt their gaze distribution in a virtual Morris water maze task based on the information that was available in the environment. Importantly, we found that compared to younger adults, older adults spent more time looking at landmarks in the *Uninformative* and *Informative Landmarks* conditions during encoding. A similar pattern was observed during the test phase in the *Informative Landmarks* condition*.*

A possible explanation for these age-related-differences in gaze behaviour is that older adults simply look around more due to a lack of a systematic encoding strategy. This can arise as a result of difficulties in selecting task-relevant information (Raptis et al., 2017). Given our results, however, it appears unlikely that older adults were randomly scanning the environment without a clear encoding strategy for several reasons: first, older adults solved the task as accurately as younger participants, which would not be possible without a clear encoding strategy. Second, we found that older adults’ gaze behaviour changed as a function of the landmarks used. Specifically, older adults spent significantly more time looking at the upper part of the stimuli when landmarks were present and when these landmarks were informative, i.e. when they could be used to encode the spatial locations of the objects by relating objects to these room-based landmarks. Third, both younger and older adults adapted their gaze behaviour over the course of the experiment such that older adults spent less time fixating on uninformative landmarks across trials. Younger participants, on the other hand, spent less time fixating on informative landmarks across the trial. These changes in gaze behaviour over time are likely to reflect adaptations of encoding strategies with older adults learning to inhibit attending to uninformative information and younger participants focusing on encoding the relationship between objects even in the presence of informative landmarks.

Finally, gaze behaviour was highly consistent between learning and test, which suggests that participants, both young and older, attended to the same information during learning and test. It is possible that low-level properties of the stimuli (i.e. colour, intensity and orientation) contributed to such similarities in gaze behaviour through bottom-up control of attention (Itti, 2005), as similar visual information was presented at both learning and test. However, given that participants performed well on the task and made very few fixations at test, it is unlikely that the consistency between gaze behaviour at learning and test was solely driven by bottom-up processes. Instead, we suggest that participants relied on the information they encoded at learning to make decisions regarding whether or not the objects have moved at test. Together, these results suggest that gaze behaviour, in both younger and older adults, represents task and stimuli-dependent visual strategies that participants employed to solve the task.

Age-related differences in gaze behaviour may also be driven by older adults being distracted by salient, but task-irrelevant landmarks as a result of attention inhibition deficits (Hasher & Zack, 1988; Healey et al., 2008, 2013). This account is partly supported by our findings as older adults spent more time than younger adults gazing at the uninformative landmarks. Notably, however, this did not affect their performance and can be explained by the relatively long encoding times that allowed participants to encode adequate task-relevant information even if they were briefly distracted.

An alternative explanation as to why older adults attended to uninformative landmarks (i.e. task-irrelevant information), is a more general age-related shift in the way they approach cognitive tasks. Zimmerman et al. (2011) suggested that older adults tend to implicitly encode all of the available information, regardless of its immediate utility. This is consistent with evidence showing that the inability to inhibit attention sometimes comes with benefits. Kim et al. (2007), for example, have shown that older adults display greater priming benefits when distractors on a previous task were used as primes in a problem-solving task. It is possible that the shift towards encoding irrelevant, as well as relevant information, stems from greater experience with real-world environments in which apparently task-irrelevant information often becomes relevant in the future (Kim et al., 2007; Zimmerman et al., 2011). For example, remembering extra landmarks in the environment could help to distinguish similar environments from each other. Such implicit shifts in encoding strategies may explain why older adults spent more time looking at extra information even if this information is not strictly necessary for solving the task at hand. However, such strategy shifts could lead to performance deficits in cognitively taxing situations, if older adults do not have enough resources to deal with the task at hand and if they are directing already limited resources to task-irrelevant information (Angel et al., 2016; Morcom et al., 2007; Reuter-Lorenz & Cappell, 2008).

The idea that older adults have a greater preference than young adults towards encoding strategies that incorporate all available landmarks is consistent with results from research that employs diffusion modelling. Several studies document an age-related shift towards a more conservative response strategy whereby, compared to young adults, older adults prefer to accumulate more information before making decisions (Ratcliff et al., 2006, 2004a, b; Segen et al., 2020; Spaniol et al., 2006; Thapar et al., 2003). This explanation is also supported by our findings of longer response times in older adults which could be indicative of greater cautiousness.

Alternatively, the preference for attending to landmarks during encoding could be indicative of age-related differences in spatial encoding strategies. Specifically, older adults’ may be more reliant on an encoding strategy in which they relate the positions of objects to landmarks, while younger participants focus on the local arrangement of objects and encode the spatial relationships between them. This interpretation is in line with our findings that older adults spent more time than younger adults looking at the landmarks during encoding, especially when these were informative. The differences in encoding strategies may represent an age-related shift towards the use of a categorical encoding strategy whereby participants bind an object to the nearest cue/landmark without the need to encode the exact metric relationship between the two. This shift may arise from difficulties in forming precise spatial representations. For example, previous visuospatial working memory research has shown that older adults were less precise in estimating previous locations of objects compared to younger adults, despite positioning the objects in the correct region of the stimuli (Nilakantan et al., 2018; Pertzov et al., 2015). Furthermore, in navigation, older adults show greater preference towards the use of beacon strategies (Wiener et al., 2013). Such strategies involve coarse categorical representations of locations in relation to environmental beacons or landmarks and may be preferred by older adults due to difficulties in formulating more precise representations.

Lastly, we did not find any relation between gaze behaviour and performance. This is not surprising as we found similar performance across different room types and across both age groups despite the presence of gaze differences. These results indicate that the current task can be solved equally well by focusing on objects and by relating the objects to landmarks (if they are available), with older adults showing a preference towards the latter. In addition, the lack of correlation between gaze and performance is consistent with our previous findings showing that the *Swap* condition could be solved either by looking around more or by having more focused gaze (Segen et al., 2020) outlining that coarse spatial representations can be formed using a wider range of encoding strategies and the available information.

To summarise, our results suggest that under specific conditions such as the presence of a relatively small perspective shift and the introduction of categorical changes within the scene, spatial memory is resistant to age-related changes as older adults perform the task as well as younger participants. Furthermore, we report an age-related shift in visual encoding strategy. Although we cannot completely rule out that these changes in gaze behaviour are driven by inhibitory control mechanisms, it seems highly plausible that older adults, who might be more distracted by the uninformative landmarks, employ an encoding strategy that relies on processing the categorical relationships between objects and room-based landmarks rather than forming fine-grain spatial representations.

## Supplementary Information

Below is the link to the electronic supplementary material.

Supplementary file1 (DOCX 438 KB)
