# Supplementary material for: Age-related changes in visual encoding strategy preferences during a spatial memory task
Source: Psychol Res. 2021 Mar 23;86(2):404–20. doi: 10.1007/s00426-021-01495-5 (PMC8885492; doi:10.1007/s00426-021-01495-5)
Supplement: Supplementary file 1 — Supplementary file1 (DOCX 438 KB) [file 426_2021_1495_MOESM1_ESM.docx]

## Supplementary Materials

**Effect Plots for Accuracy Analysis**

**
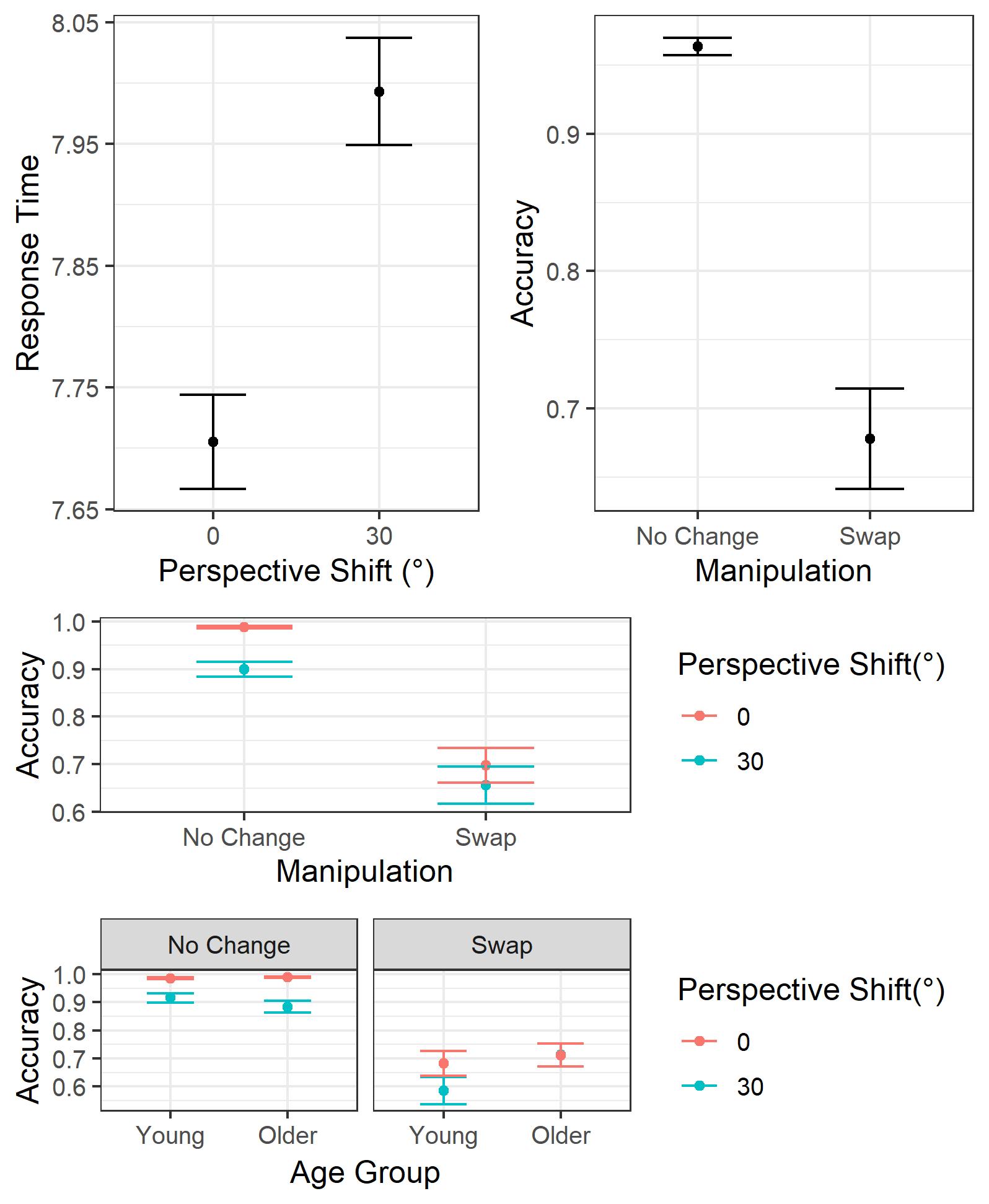
**

Figure 1. LMM Effect plots for significant main effects and interactions for performance accuracy

**Effect Plots for Response Time Analysis**

**
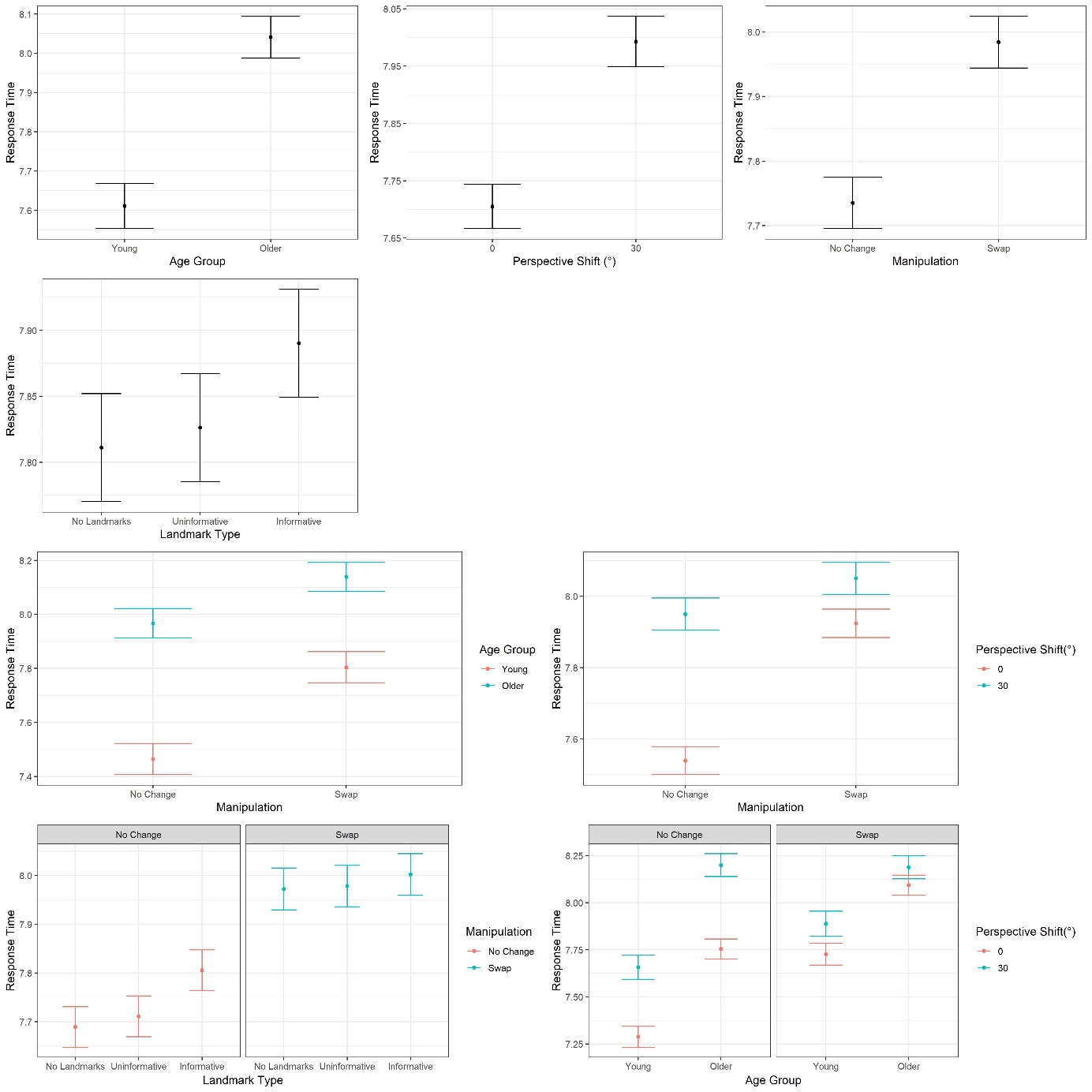
**

Figure 2. LMM Effect plots for significant main effects and interactions for response times

**Basic Fixation and Saccade Parameters.**

Differences in basic fixation and saccade parameters were examined using the Bootstrap-t method (5000 resampling) with 20% trimmed means (Wilcox & Keselman, 2003). This method provides a robust estimation of central tendency as it reduces the probability of Type 1 error and bias and does not compromise power as median-based methods do (Wilcox & Keselman, 2003). During the 12 second encoding period, older adults made shorter and more frequent fixations as well as more frequent saccades (see Table 1). Note however that these variables are not independent of each other especially in a fixed time interval.

Table 1 Means and t-test results for saccade and fixation parameters between younger and older adults from the Learning Phase

| Gaze measure | Mean Young | Mean Old | Confidence Interval | t-value | p-value |
| --- | --- | --- | --- | --- | --- |
| Saccade Frequency | 2.51 | 2.84 | [-0.55, -0.11] | -2.94 | **.004** |
| Average velocity | 138.98 | 134.85 | [-9.51, 17.77] | -0.62 | .524 |
| Peak velocity | 252.01 | 246.81 | [-21.23, 31.63] | 0.40 | .697 |
| Amplitude | 6.81 | 6.41 | [0.46, 1.25] | 0.94 | .337 |
| Saccade duration (ms) | 39.36 | 39.58 | [-2.59, 2.13] | -0.20 | .844 |
| Fixation Frequency | 2.67 | 3.11 | [-0.67, -0.21] | -3.74 | **.001** |
| Fixation Duration (ms) | 286.23 | 255.88 | [10.98, 50.77] | 3.10 | **.005** |
| *Note: significant p values are in* ***bold type*** | | | | | |

### Gaze Behaviour at Test

As in the encoding phase of trials, an LME analysis with Age Group and landmark Type as fixed factors and by-subject and by-item random intercepts in the test phase showed that landmark Type was predictive of Dwell Time on landmarks (Table 2). Specifically, we also found an Age Group and landmark Type interaction with older adults showing a larger increase in Dwell time on *Informative* compared to *Uninformative landmarks* compared to younger adults. Interestingly, at test older adults no longer displayed a larger increase in Dwell Time compared to younger adults between the *No landmarks* and *Uninformative landmarks* conditions, suggesting that they may have realised that *Uninformative landmarks* were not useful for solving the task.

Table 2 Coefficients from Dwell Time on the top IA at Test LME analysis

|  | **Dwell Time** | | |
| --- | --- | --- | --- |
| *Predictors* | *Estimates* | *std. Error* | *t-value* |
| (Intercept) | 7.939 | 1.311 | 6.058 |
| Age Group (*Older Adults*) | 1.899 | 1.262 | 1.505 |
| landmark Type (*No landmarks*) | -4.002 | 0.682 | **-5.868** |
| landmark Type (*Informative*) | 10.798 | 0.682 | **15.839** |
| Age Group (*Older Adults*): landmark Type (*No landmarks*) | -0.872 | 0.462 | -1.889 |
| Age Group (*Older Adults*): landmark Type (*Informative*) | 3.132 | 0.461 | **6.791** |

Significant t values (|t|≥1.96) in **bold**
